# Celiac disease with a mixed pattern: a case report

**DOI:** 10.1186/1757-1626-2-9330

**Published:** 2009-12-16

**Authors:** Yalcin Basaran, Ismail Simsek, Armagan Gunal

**Affiliations:** 1 Department of Internal Medicine, Gulhane Military Medical Academy, Tevfik Saglam Street, Ankara, 06010, Turkey; 2 Department of Rheumatology, Gulhane Military Medical Academy, Tevfik Saglam Street, Ankara, 06010, Turkey; 3 Department of Pathology, Gulhane Military Medical Academy, Tevfik Saglam Street, Ankara, 06010, Turkey

## Abstract

**Introduction:**

Celiac disease can be severe and associated with progressive malabsorption and death. A subset of patients may develop subepithelial collagen deposition, a condition referred to as collagenous sprue.

**Case presentation:**

We report a case of a 46-year-old female who was previously diagnosed as having seronegative arthritis and inflammatory bowel disease, and three years later after the initial diagnosis she was histologically confirmed to have celiac disease in association with collagenous sprue, another underlying malabsorptive disorder.

**Conclusion:**

Although the precise relationship between celiac disease and collagenous sprue has been debated and remains controversial, it should be considered among the differential diagnoses of chronic diarrhea with progressive malabsorption.

## Introduction

Celiac disease (CD) is a disorder characterized by mucosal inflammation and villous atrophy of small bowel, leading to symptoms of malabsorption such as steatorrhea, weight loss or other signs of nutrient or vitamin deficiency. Withdrawal of gluten-containing foods results in resolution of the mucosal lesions and symptoms. The diagnosis is presumptively established when there is concordance between the serologic results and the biopsy findings. It is confirmed when symptoms resolve subsequently on a gluten-free diet.

Collagenous sprue (CS) is a rare disease of the small bowel, which is characterized by complete atrophy of mucosal villi and excessive collagen deposition in the lamina propria. It may cause a life threatening clinical picture. Patients may also present with a number of non-gastrointestinal manifestations; neuropsychiatric, rheumatic and metabolic disorders. Patients with CS generally do not respond to a gluten-free diet and often have a poor prognosis, but luckily some of them experience complete clinical improvement.

## Case presentation

A 46-year-old Kirghiz female was admitted with the previous diagnosis of seronegative arthritis and inflammatory bowel disease. Various nonsteroidal anti-inflammatory drugs, prednisone, sulfasalazine, and two courses of anti-TNF alpha were prescribed earlier by her physician for symptomatic treatment.

At admission to our clinic, she presented with depressive symptoms and anxiety. She complained of weight loss (loss of 15 kg over a period of 1 to 2 months), diarrhea, weakness, and fatigue. She was severely cachexic with a significantly lower BMI (12.11 kg/m^2^). Physical examination revealed bilateral swelling of knee joints, skin rashes over the lower legs and the presence of pitting after applying pressure to pretibial areas. Movements of involved joints were painless and there was no definite restriction in the range of movements. The liver was palpated 1 cm below the costal margin in the right upper quadrant. Splenomegaly and enlargement of lymph nodes were not noticed.

Complete blood count at admission showed the following values: Hb: 11.2 g/dL, Hct: 32.6%, WBC: 7.500/mm^3^, Plt: 351.000/mm^3^. The erythrocyte sedimentation rate was 4 mm/h, and the level of C-reactive protein was 13.6 mg/L. The routine biochemical tests revealed hyponatremia (126,6 mmol/L, normal: 135-145), hypokalemia (2.59 mmol/L, normal: 3.5-5.5), low ionized calcium (3.18 mg/dL, normal: 4.2-5.4), magnesium (1.37 mg/dL, normal: 1.9-2.7) and phosphate concentrations (1.2 mg/dL, normal: 2.6-4.5), severe hypoproteinemia (4.78 g/dL, normal: 6.4-8.3), hypoalbuminemia (1.2 g/dL, normal: 3.5-5.5), elevated levels of alkaline phosphatase (298 U/L, normal: 38-155) and gammaglutamyl transpeptidase (127 U/L, normal: 7-32). The other biochemical results were within normal limits and routine urine examination was normal. The patient had a 24-hour urinary protein collection, which showed a 24-hour protein measurement of 67.50 mg. This was confirmed on a repeat sample. Thyroid function tests showed FT3 level of 0.62 pg/mL at the lower limit of the normal range (normal: 0.60-1.95), decreased FT4 level of 4.40 ng/dL (normal: 5.00-11.50) and normal TSH concentration of 1.14 μIU/mL (normal: 0.30-4.00). The concentrations of PTH (230 pg/mL, normal: 10-65) and 25-hydroxyvitamin D (4 ng/mL, normal: 10-40) were also measured. Electrophoresis of alkaline phosphatase isoenzymes revealed elevated levels of liver isoenzyme. To view the status of hypothalamic-pituitary-adrenal function synachten test was performed, which excluded the possibility of adrenal insufficiency. The serological tests for various infectious agents (HBsAg, anti HBC, anti HIV 1+2, Treponemal tests, Brucella Ig G/M antibody levels, TORCH titers) were found to be negative. Ig G, A, M levels were within normal limits. AMA, c-ANCA, p-ANCA, anti-LKM-1 were not detected. She was found to have positive ANA in a titer of 1/80 with granular and homogeneous pattern. Positive IgA endomysial antibody (1/80 titer) and IgG antigliaden antibody (98.50 RU/ml) levels were also detected.

Radiographs of involved joints revealed no evidence of bony erosions, chondrocalcinosis or deformity. Findings on electrocardiogram, echocardiogram, and chest radiograph, and magnetic resonance cholangiopancreatography images were all normal. Abdominal ultrasonography displayed enlarged liver (155 mm in maximum cranial-caudal extent) and increased echogenicity of the liver parenchyma (Grade 1). On thoracoabdominopelvic computed tomographic examination multiple mesenteric lymph nodes, with a largest diameter of 18 × 10 mm, were detected. DEXA scan of the lumbar spine and right hip showed low bone mineral density (BMD) with a total T score of - 5.7 and 4.9, respectively.

**Figure 1:**
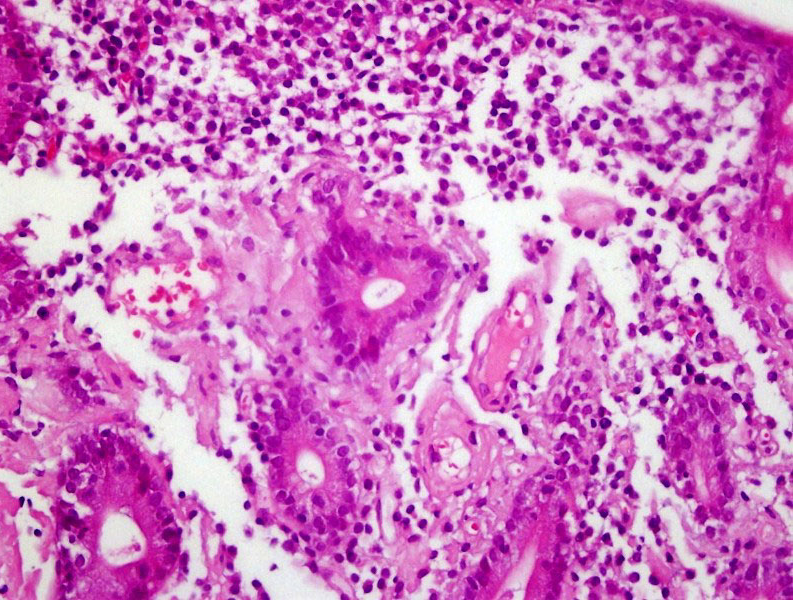
**Eosinophilic material deposition in the lamina propria (Hematoxylin and Eosin staining, 200× magnification)**.

**Figure 2:**
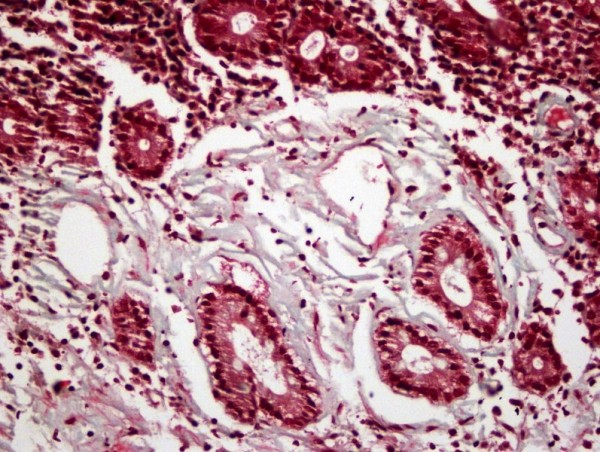
**Light-green staining of the deposition (Trichrome staining, 200× magnification)**.

To examine the upper part of the gastrointestinal system esophagogastroduodenoscopy was done, which revealed grade 1 esophagitis, hypotonic LES, gastritis, erosive bulbitis and hyperemia in the postbulbar duodenum. To confirm the initial diagnosis of inflammatory bowel disease colonoscopy was performed. Findings other than nonspecific hyperemia of the ileal mucosa were not present. Multiple biopsies were obtained from both upper and lower gastrointestinal tract at the time of endoscopic evaluation. CD was histologically confirmed by duodenal biopsies, which revealed total villous atrophy of duodenal mucosa and regenerative hyperplasia. Increased cellularity of plasma cells and lymphocytes in the lamina propria and surface epithelium were observed. Histological examination of the excised tissue also revealed amorphous eosinophilic deposition in the lamina propria and in the wall of some vessels and glands, which was previously found typical for amyloid (Figure 1). However, congo-red and crystal violet staining prosedures and immunohistochemical studies showed no amyloid protein. Neither kappa nor lambda monoclonality was detected in lymphoplasmocytoid cells, immunohistochemically. Loose light-green staining of the deposition by trichrome staining was the unique positive finding (Figure 2). In the light of these findings, collagenous sprue due to long-standing celiac disease was the last diagnosis, histologically. Specimens obtained from terminal ileum revealed chronic inflammation and subtotal villous atrophy of ileal mucosa.

Our patient suffered from anorexia and poor oral intake. To increase caloric intake to gain weight total parenteral nutrition was administered by cyclic infusions. After several days diarrhea improved. She was asked to avoid taking gluten and her complaints abated and did not persist.

## Discussion

The combination of a careful history and physical examination and a few diagnostic tests (serologic results and the biopsy findings) can result in establishing the diagnosis of CD and CS in the majority of patients. CD is confirmed when a rapid response to a gluten-free diet is achieved [1], however patients with CS generally do not respond to a gluten-free diet [2]. In our case, the patient was diagnosed with CD based upon serological and histological findings with compatible clinical and laboratory manifestations and noticeable clinical improvement was observed on a gluten-free diet within several days.

CD may be associated with arthritis in some patients [3]. Articular involvement is peripheral in 19 percent, axial in 15 percent, and combined in 18 percent. The arthritis is typically nonerosive and can be either oligoarticular or polyarticular. Joint symptoms may precede gastrointestinal manifestations of the disease and respond to a gluten free diet. Our patient had been misdiagnosed to have seronegative arthritis and had not been accurately treated since several years.

Bone loss (principally osteopenia and less often osteoporosis) is common in CD [4] and much of the bone loss is related to secondary hyperparathyroidism, which is probably due to vitamin D deficiency [5]. A low serum 25(OH) D concentration is the hallmark of vitamin D deficiency, which leads to hypocalcemia and hypophosphatemia. Hypocalcemia stimulates the release of PTH, resulting in increased effect of PTH on bone. PTH also enhances urinary phosphate excretion and contributes to the development of hypophosphatemia. The biochemical laboratory studies of our case were consistent with the diagnosis of secondary hyperparathyroidism and she had significantly decreased BMD in the lumbar spine and femoral neck. Calcium and vitamin D supplements were initiated to reduce the risk of bone loss and fractures and to enhance the mineralization of bone. Additionally, the patient was started on once-weekly bisphosphonate therapy after her dyspeptic complaints abated.

It has previously been shown that CD may be associated with neuropsychiatric symptoms such as depression or anxiety [6]. Our case presented with prominent anxiety and episodic headaches, and she was started on an anxiolytic medication.

Increased prevalence of coexisting thyroid disease (usually hypothyroidism and less often hyperthyroidism) has been reported among patients with celiac disease [7]. In our patient, serum TSH levels did not increase despite the low FT4 and low-normal FT3 values.

Our patient presented with weight loss, diarrhea, weakness, and fatigue. On physical examination, cachexia and pretibial edema were detected. Laboratory studies revealed hypoalbuminemia with normal daily protein excretion of less than 150 mg and elevated levels of alkaline phosphatase and gammaglutamyl transpeptidase. Abdominal ultrasonography revealed hepatomegaly and increased echogenicity of the parenchyma. These symptoms and findings may all be suggestive of the contribution of gastrointestinal amyloidosis to malabsorption [8], protein-losing gastroenteropathy [9] and probably to hepatic involvement [10]. Additionally, histological examination of duodenal mucosa was consistent with diagnosis of amyloidosis, but further immunohistochemical studies showed no amyloid protein.

## Conclusion

Although, CD is rarely reported to be associated with CS, it should always be in mind as a possible cause for symptoms. This relationship is rare but it should be considered among the differential diagnoses of chronic diarrhea with progressive malabsorption and clinical follow-up in specialized centers is warranted in the management of these cases. Patients with CS are generally considered to be gluten insensitive and often to have a poor prognosis, but luckily gluten sensitivity differs from patient to patient and it may, in some instances, be a completely reversible small intestinal disorder.

## Abbreviations

CD: Celiac Disease; CS: Collagenous Sprue

## Consent

Written informed consent was obtained from the patient for publication of this case report and accompanying images. A copy of the written consent is available for review by the Editor-in-Chief of this journal.

## Competing interests

The authors declare that they have no competing interests.

## Authors' contributions

This report reflects the opinion of the authors and does not represent the official position of any institution or sponsor. YB analyzed and interpreted the patient data, ÝS contributed to the final draft of the manuscript and analysis of relevant data, AG performed the histological examination and was responsible for preparing photographs. All authors read and approved the final manuscript.
